# Factors Affecting Gastrointestinal Microbiome Development in Neonates

**DOI:** 10.3390/nu10030274

**Published:** 2018-02-28

**Authors:** Clara Yieh Lin Chong, Frank H. Bloomfield, Justin M. O’Sullivan

**Affiliations:** 1 Liggins Institute, The University of Auckland, Private Bag 92019, Auckland 1142, New Zealand; clara.chong@auckland.ac.nz (C.Y.L.C.); f.bloomfield@auckland.ac.nz (F.H.B.); 2 Newborn Services, Auckland City Hospital, Auckland 1023, New Zealand

**Keywords:** in utero development, microbiota, vaginal birth, Caesarean-section, infant feeding, Intrapartum antimicrobial prophylaxis, Neonatal Intensive Care Unit, Human milk oligosaccharides

## Abstract

The gut microbiome is established in the newborn period and is recognised to interact with the host to influence metabolism. Different environmental factors that are encountered during this critical period may influence the gut microbial composition, potentially impacting upon later disease risk, such as asthma, metabolic disorder, and inflammatory bowel disease. The sterility dogma of the foetus in utero is challenged by studies that identified bacteria, bacterial DNA, or bacterial products in meconium, amniotic fluid, and the placenta; indicating the initiation of maternal-to-offspring microbial colonisation in utero. This narrative review aims to provide a better understanding of factors that affect the development of the gastrointestinal (GI) microbiome during prenatal, perinatal to postnatal life, and their reciprocal relationship with GI tract development in neonates.

## 1. Introduction

Humans are holobionts, a complex ecosystem of host-derived cells together with transient and stable microbial symbionts [1]. A typical human body contains up to 100 trillion microorganisms, equivalent to ~10 times the total number of nucleated cells in the body [2,3,4,5]. The large intestine is the greatest single human reservoir of microbes, containing at least 30 identified genera and as many as 500 different species [2,6,7]. The inter-relationships that occur within this ecosystem are complex and affect the development and health of the individual [8].

The association between the development of the gut microbiota and the host’s genotype and phenotype has received increasing attention as technological advances in culture-independent techniques (e.g., genomic, transcriptomic, proteomic, and metabolomic) have facilitated the detection of a greater diversity of microbes [9]. These studies have demonstrated that the composition of the gut microbiota in each infant is idiosyncratic with significant inter-individual variation being evident from the first day after birth [10,11,12]. Individuality, time from birth and mode of feeding were the strongest contributors to variation in the microbiota in 8 infants sampled seventeen times over the first 12 weeks from birth [13]. Notably, the impact of individuality on microbiota development was more pronounced in breast-fed babies consistent with the impact of fluctuations in environmental effects (e.g., mother-specific fluctuations in milk composition) [13]. The infant’s gut microbial composition increases in number and diversity as they age [14,15]. By around three years of age, the infants’ gut microbiota will attain a diversity and complexity of composition that resembles the mature adult anaerobic gut microbiota [4,14,16,17].

It is clear that under “normal” circumstances, the gut microbiota has a symbiotic relationship with the host during which, among other things, it contributes to: the storage and harvesting of energy [18]; development of the host immune system [6,19,20,21]; maintenance of intestinal homeostasis [22]; and, nutrient processing [12]. Interactions between gut microbes and the host also have a profound effect on an individual’s health later in life [23], while perturbation of the gut microbiota population structure (i.e., dysbiosis) is associated with pathological conditions [24] that include inflammatory bowel disease (IBD) [25], obesity, allergic [26], and autoimmune diseases [27].

**Figure 1:**
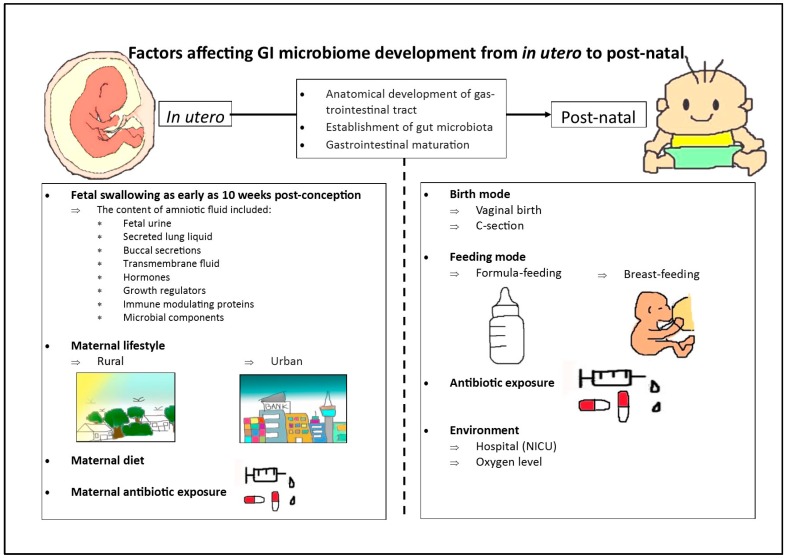
Factors from in utero to post-natal life that have been shown to affect the establishment of the gastrointestinal (GI) microbiome.

Despite our awareness of the significance of maintaining the mutual relationship between the host and gut microbiota across the life-span, conclusive evidence of the factors that affect the development of the microbiome are not yet available. Various factors have been proposed to affect the early-life development of the microbiota, including: the composition of the maternal microbiome [28]; mode of birth [10,24,29]; antibiotic usage [30]; and, length of gestation [31,32] (Figure 1). In this review, we will focus on factors that are known to affect the establishment of the biggest human microbial reservoir—the GI tract and the reciprocal relationship between GI microbiota and GI tract development from in utero to post-natal life.

## 2. Development of the GI Tract

The human gastrointestinal tract or alimentary canal starts from the mouth, extending through well-defined anatomical regions—the oesophagus, stomach, small intestine, colon, rectum—and ending at the anus [6]. The functional and structural development of the GI tract is a crucial part of human development as the gut must accommodate the diversity of dietary inputs and foreign antigens that are introduced into the human body together with food throughout different stages of life [33]. 

The maturation of the human GI tract starts in utero but continues after birth with some functions, such as epithelial barrier mechanisms, accessory structures (e.g., glands), and the intestinal immune system, only becoming fully developed several months or years after birth [33]. The primitive gut forms from the dorsal section of the yolk sac approximately 22 days after conception, leading to the appearance of the foregut, midgut, and hindgut approximately 25 days after conception [34]. The stomach appears approximately five weeks post-conception. The midgut rapidly increases in length until it can no longer fit within the developing abdominal cavity and herniates into the vitelline sac before undergoing complex rotations and returning to the abdominal cavity after approximately 10 to 12 weeks of gestation [34]. GI tract development continues until all the major tissue components of the mature gut are present after approximately 20 weeks’ post-conception [33]. Despite the fact that the GI tract originates from the dorsal section of the yolk sac, there are regional-specific tissue features (e.g., gastric pits, glands villi, and crypts) that differentiate between the different sections of the gut [33].

Comparative studies have identified abnormalities in gut-associated lymphoid tissue development and decreased antibody production in germ-free mice [35]. Similarly, germ-free piglets have been shown to have alterations to their intestinal physiology that include reduced turnover of the intestinal epithelial cell and a reduction in mucosal biosynthetic rate when compared to control animals [36,37]. Collectively, these findings are consistent with the structural and functional development of the GI tract being affected by the composition and the activity of an individual’s microbial flora. Because of the nature of GI tract development, these microbe-specific effects are likely to be due to impacts on developmental processes that occur both in utero and postnatally. 

## 3. Microbial Impacts on In Utero GI Development 

We have long assumed that the gut is sterile before birth [38,39,40,41,42]. However, this dogma was challenged when several studies identified bacteria, bacterial DNA, or bacterial products in meconium [10,43,44], amniotic fluid [23,45], and the placenta [23,46]. Yet, evidence of live bacterial culture from placental and amniotic fluid samples remains limited [23]. Despite the limited evidence, these findings raise the possibility that intrauterine human gut development that prepares the protective barrier necessary for enteral feeding after birth is affected by the development of stage-specific microbiota that begins in utero [23].

## 4. The Importance of Fetal Swallowing for GI Development

In order for the microbiome to affect GI development in utero, there must be a mechanism to ensure the selection and exposure of the appropriate microbial population or factors. The obvious medium for such a system is the amniotic fluid that bathes the developing fetus. Notably, the composition of amniotic fluid varies over the course of gestation [47]. Amniotic fluid is composed primarily of fetal urine, with contributions from secreted lung liquid, buccal secretions, and transmembrane flow [33]. Amniotic fluid also contains hormones and growth regulators [48], immune modulating proteins, and microbial components [23]. It remains unclear how the selection of particular microbes would be achieved within the amniotic fluid; however, interactions between typical environmental factors (e.g., pH, oxygen levels, carbon sources), innate and learned immunity are obvious candidates. 

One of the potential route for the exposure of the developing GI to microbes/microbial products within the amniotic fluid can be mediated by swallowing. The human fetus begins swallowing amniotic fluid as early as 10 weeks post-conception [48]. This coincides with the oesophagus being innervated, which typically occurs by 13 weeks [33]. In the last trimester of pregnancy, the human fetus swallows between 700 and 1000 mL amniotic fluid per day [49,50]. This provides a possible medium by which bacteria and bacterial products (e.g., glycoproteins, RNA and DNA) can be introduced into the developing gut. 

Data from human infants born with gut abnormalities, such as gastroschisis and intestinal atresia, suggest that despite nutrition being obtained through the placenta, fetal gut development affects fetal growth in utero [50]. Babies with a proximal gastrointestinal atresia have reduced birthweight compared to babies born with a distal atresia [50,51] and term babies with atresia have significantly reduced birthweight centiles compared with those born prematurely with atresia [50]. These data are consistent with observations from animal studies, which also have demonstrated the trophic effect of amniotic fluid [52] and that fetal swallowing is essential for normal development of the gut [53]. For example, fetal sheep that have undergone oesophageal ligation exhibit reductions in the thickness of the external muscle layers in their stomach, duodenum and proximal small intestine, changes in the length of intestinal villi, and altered rates of epithelial cell migration [34]. In rabbits that had undergone oesophageal ligation, infusion of amniotic fluid beyond the ligation resulted in relatively normal gut development as compared to that of rabbits with an oesophageal ligation alone [52]. These findings reveal the importance of swallowing amniotic fluid in utero for the anatomical development of the GI tract. 

It remains to be determined if it is the act of swallowing or specific components of the amniotic fluid that are responsible for the observed impacts on development. However, oral administration of labelled *Enterococcus fecium* to pregnant mice resulted in this organism being isolated from the meconium of the newborn pups but not from the control group [44], as consistent with a mechanism for translocation to the amniotic fluid for fetal ingestion. Consistent with this, additional studies have demonstrated that maternal microbes can be transported to the amniotic fluid [23,44] and the placenta [54]. However, irrevocable evidence for intrauterine transmission of microbes from mother to fetus that results in active colonisation and developmental impacts is still lacking. 

## 5. Factors Affecting GI Microbiome Development after Birth

The complexity and richness of the microbial community progress through a series of developmental stages that range from the neonatal period before there is an apparent stabilisation after weaning. There are several crucial interrelated factors, together with individuality, which play an important role in shaping the transitioning human GI microbial composition. These factors include age [55,56], diet [24,29], host genetics [24,29,57], antibiotic usage [24,29,56], the physiology of the colonisation site [6], mode of birth [10,24,29], type of feeding (i.e., breast milk or formula milk) [10,24], and the birth environment of the infants (e.g., NICU) [10].

**Figure 2:**
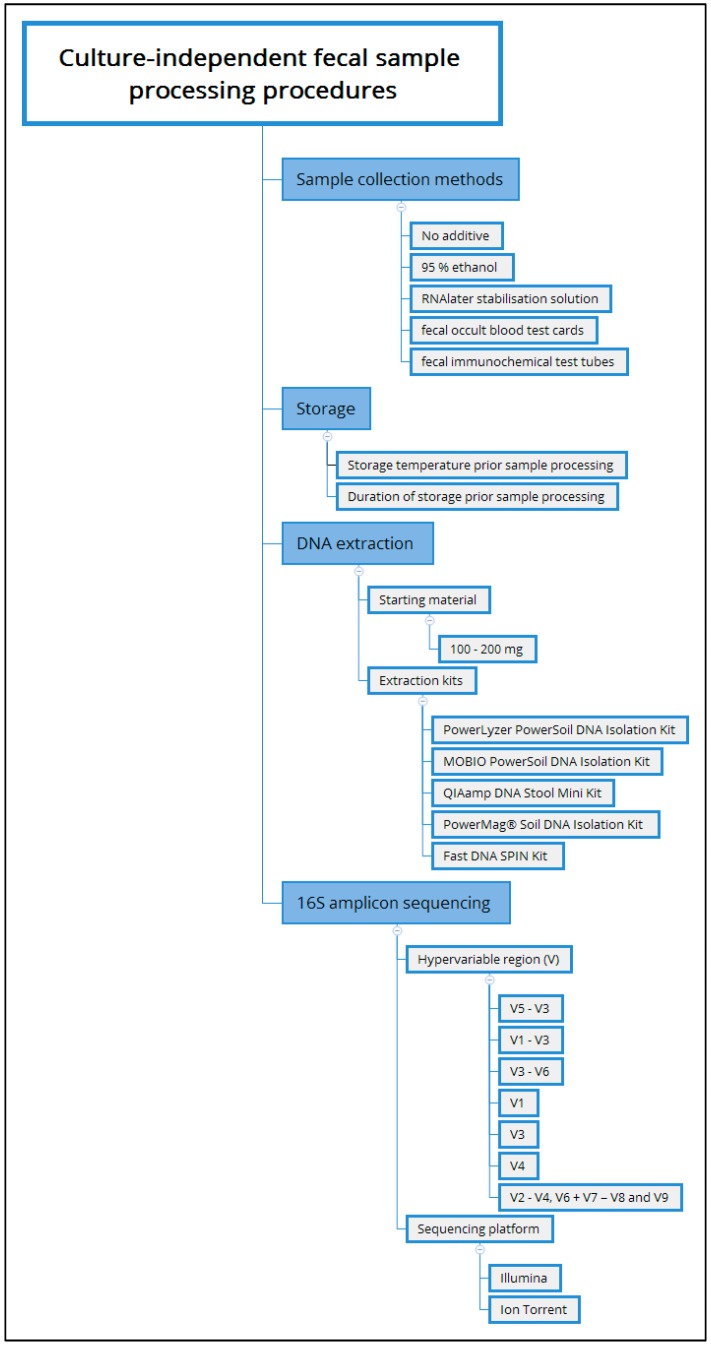
Variables that can impact on culture independent approaches for fecal microbiome characterisation.

The shape of the developing microbial composition is also affected by technical variation (Figure 2). For example, culture based techniques for the identification of microbes are subject to biases that arise from: (1) oxygen-sensitivity; (2) the recalcitrance of some bacterial species to culture media; and, (3) competition between fast- and slow-growing bacteria. This limits current culture-based methods to the successful isolation of no more than 70% of intestinal microbes in a sample when compared to culture independent approaches [58].

Culture-independent techniques, typically use high throughput sequencing or array technologies to analyse the extracted nucleic acids from the community. These techniques are subject to variation due to processing, the kits that are used to prepare the DNA for analysis, and the computational analyses themselves. Commercially available kits including the PowerLyzer PowerSoil DNA Isolation Kit (MoBio, Carlsbad, CA, USA) [43,59], QIAamp DNA Stool Mini Kit (QIAgen, Hilden, Germany) [23], MOBIO PowerSoil DNA Isolation Kit (MOBIO) [27], PowerMag^®^ Soil DNA Isolation Kit (MO-BIO Laboratories, Inc., Carlsberg, CA, USA) [60], and Fast DNA SPIN Kit (MP BIO, Santa Ana, CA, USA) [9,58] have been widely used. 

Despite the use of commercial kits for DNA preparation, sample processing methods, including different amounts of starting material (e.g., 100 mg to 200 mg [43,59]), also introduce potential biases. Sample storage (i.e., duration and temperature) prior to processing varies (e.g., store at 4 °C and process within 24 h [27] and 72 h [61]; snap-freeze in liquid nitrogen; or place on dry ice immediately following collection and store at −80 °C [43])and contributes to potential biases in the sample composition. Inter-study variation is further compounded by the use of different 16S rRNA gene hypervariable regions (e.g., V5 − V3 [27]; V1 and V3 [9]; V1 − V3 [23]; V3 − V6 [13]; V2 − V4, V6 + V7 − V8 and V9 [62] and the most frequent amplified region—the V4 region [43,59,60]) which also contributes to variation between different studies. It is important to keep these systematic biases in mind as we consider the variation between studies. 

### 5.1. Influence of Birth Mode on the Establishment of GI Microbiota

The mode of birth determines the microbial population that infants are exposed to during birthing. For instance, vaginal birth exposes infants to the microbes that are currently colonising the mother’s birth canal. This direct form of inheritance during birth results in infants who are born by vaginal delivery having a similar microbiota to that of their own mother when compared to other mothers [38,63]. By contrast, significant overlap between the microbiota of mothers and children that are born by C-section has not been observed [38,63]. Rather, environmental factors (e.g., delivery and surgical equipment, air, other infants and healthcare workers) appear to have a greater effect on the microbiome of infants born by C-section [6,61]. Recent findings for infants born by C-section indicated that a period of labour prior to the surgery was associated with infants having a microbiota that more closely resembled that of vaginally-born infants, whereas infants born without any labour period had a microbiota that resembled that of the maternal skin [27]. C-section is suggested to be one of the reasons for early-life microbial disruption and this perturbation in microbial colonisation during infancy affect microbial-host interaction, which can lead to long-term metabolic consequences in the host [64,65,66]. In addition, higher chances to acquire atopic diseases during the first two years after birth also demonstrated by C-section infants as compared to vaginally birth infants based on data from 2500 full-term healthy newborns in LISA-Study [67].

A good example of the effect of birth mode on the gut microbiota comes from the impact of birth mode on the acquisition of *Lactobacillus* in the infant’s GI tract. *Lactobacillus* is highly abundant in, and specific to, the maternal vagina with an IndVal index of 0.922 [27]. It has been reported that infants born through the mother’s birth canal contain *Lactobacillus* as part of their microbiome profile, but that infants born by C-section do not [65]. These early observations gained further support in a subsequent study that detected significantly fewer *Lactobacillus* genus in the microbiome profile of infants born by C-section (*n* = 17) versus vaginal (*n* = 134) (Detection rate of 6% vs. 37%) [68]. Notably, the low detection rate of *Lactobacilli* in the intestinal discharge of the C-section infants persisted for the first six months after birth in contrast to vaginally born infants, who had higher and increased detection rates throughout the first six months after birth [68]. However, this difference in *Lactobacilli* detection rates vanished by three years of age [68].

The levels of bacteria in the *Bacteroides* and *Clostridium* genera (e.g., *Bacteroides fragilis* and *Clostridium difficile*) within an individual’s microbiota are also associated with birth mode [38,39,43,61,63,69,70]. In the KOALA Birth Cohort study in the Netherlands (*n* = 1032), real-time quantitative PCR assays were used to enumerate different bacterial species from stool samples collected at one month of age [39]. Infants born by unassisted vaginal birth (*n* = 826) had relatively high numbers of *B. fragilis* and a reduced number of *C. difficile* compared to C-section infants [39]. By contrast, stool samples from infants born by C-section (*n* = 108) showed the inverse relationship [39]. The sources of *C. difficile* could be linked to environmental factors rather than the mother, as *C. difficile* was detected on the hands and in the stools from healthy hospital personnel [39,71]. Notably, *C. difficile* has been considered as a microorganism that exists exclusively in hospitals [72] and has been shown to be absent from vaginal swabs from women prior to delivery [73,74]. This may explain the high levels of *C. difficile* identified in hospital-born and C-section infants [39]. 

At the phylum level, a low abundance of *Bacteroidetes* (*p* = 0.002) was also observed in infants born by C-section (*n* = 9) when compared to vaginally born infants in a study of 24 infants conducted in south-eastern Sweden [38]. Notably, this reduction in the abundance of *Bacteroidetes* persisted for the first two years after birth [38]. This is consistent with previous reports that highlight delayed establishment of members of the *Bacteroides* in C-section infants in the first six months [61] and one year of life [75]. Notably, members of the *Bacteroides* genus are highly specific to the maternal stool (IndVal index of 0.943) [27]. Collectively, these findings highlight the central role of exposure to the maternal stool environment during birthing for the early inheritance and establishment of *Bacteroides* in the infant’s microbial profile. 

Not all studies have found an association between mode of birth (vaginal versus C-section), the inheritance and development of the GI microbiota. For example, a study of 21 infants found that mode of birth did not affect microbial population in preterm babies throughout the first three months after birth [11]. The incorporation of preterm infants (gestational age of 30 to 35 weeks) in this study could potentially explain this, as another study also found that birth mode was not significantly associated with microbiome composition in preterm infants [59]. In summary, studies indicate that infants born by C-section tend to have: lower numbers of anaerobes (e.g., *Bacteroidetes*); a less diverse microbiota [21,38,61]; delayed colonisation of microbial population [43]; and, they acquire atopic diseases [21] and metabolic disorders [66] more frequently than infants born by unassisted vaginal birth. However, these studies are complicated by ethnic and geographic diversity and differences in analytical methodologies. 

### 5.2. Impact of Feeding on GI Microbiome Development 

Milk is the first food that is introduced into the GI tract postpartum and the composition of the milk is believed to directly impact on shaping the early GI microbiota [3,4]. This impact can occur through the provision of: essential nutrients for bacterial proliferation [3]; immunomodulatory molecules [76]; and, microbes that are capable of colonising the infant [77]. The possibility that feeding type contributes to the early post-natal development of the GI flora is supported by an observed similarity between microbial composition in colostrum and the meconium from infants who were breast-fed from the first hour after birth [23]. Shared bacterial DNA (e.g., homologous to *Streptococcus thermophilus*, *Staphylococcus epidermidis*, and *Bifidobacterium longum*) has also been identified in human breast milk and infants’ faecal samples [78]. This relationship is more pronounced between infants, their mother’s milk and areolar skin when compared to a random mother (*p* < 0.001) [14]. Furthermore, *Bifidobacterium longum* rDNA sequences can be co-detected in infant’s faeces, maternal blood, maternal faeces and breast milk collected between one and four weeks post-birth [78]. Collectively, these results are consistent with the breast milk mediated vertical transfer of microbial communities to the infant’s gut [14]. 

Culture-based techniques have identified more diverse microbiomes in formula-fed infants when compared to breast-fed infants [79]. Culture-independent studies have supported this observation [9,80]. For example, Lee et al. (2015) characterised the effect of feeding type on the microbiota of 20 Korean infants who were born vaginally at term to healthy mothers [9]. Faecal samples were collected at four weeks of age from 10 exclusively breast-fed and 10 formula-fed infants. Relatively small amounts of formula supplementation (once every 24 h during the first week after birth) to breast-fed infants shifted the microbial profile towards a pattern that was similar to that observed for exclusively formula-fed infants [81]. Although some formula-fed infants were exposed to breast milk, these infants were fed with a diet that consisted of 70–100% formula milk [9]. Five species of bacteria were present in the faecal samples of all the infants in this study (i.e., both breast- and formula-fed group contained *Bifidocbacterium longum*, *Streptococcus salivarius*, *Strepotococcus lactarius*, *Streptococcus pseudopneumoniae*, and *Lactobacillus gasseri*). Lee et al. (2015) argued that the presence of these species in the intestines of these babies must be independent of feeding type, and thus these species constitute common commensal bacteria that are present in four-week-old Korean infants [9]. However, the relative abundances of these species differed in both groups, with *B. longum*, *S. pseudopneumoniae*, and *L. gasseri* having a greater abundance and *S. salivarius*, and *S. lactarius* having a lesser abundance in breast-fed infants when compared to formula-fed infants. These results are consistent with the hypothesis that the relative abundance of common commensal bacteria is altered by exposure to different feeding patterns, formula or breast milk.

Collectively, formula-fed infants tend to have relatively stable and diverse GI microbial communities that contain higher levels of facultative anaerobes and strict anaerobes when compared to breast-fed infants [9,37,82,83]. Faecal samples from breast-fed infants are less complex, contain higher numbers of aerobic organisms, and exhibit more dramatic changes in microbial composition over their first year after birth [9,82,83]. Studies suggest that, once weaning (i.e., the introduction of solid foods into the diet) starts, the differences in microbial population between breast and formula-fed infants are lost and the microbial communities converge towards a complex adult microbiome [3,6]. However, a recent study (*n* = 107) reported that the continuation of breast milk feeding after the introduction of solid food suppresses the diversification of the microbiota associated with the introduction of solid food [14]. The effect and mechanism of this suppression is yet to be determined and more studies that allow for other factors, such as ethnicity of subjects, to be controlled are required. 

#### Nutrients and Microbial Composition of Human Breast Milk

The nutritional composition of human milk varies as lactation progresses. Besides nutrients, breast milk also contains hormones [37], growth factors [37], microbiota [14,84], immunoglobulin [37,85], and enzymes [37,85]. The protein content of early human milk from mothers who gave birth to preterm children is higher than that from mothers who gave birth at term [86,87]. This is partly explained by a higher demand for protein to support growth in preterm infants compared to infants born at term. However, this protein content declines steadily with lactation [88] and is negatively associated with milk volume output at six and nine months after birth [89].

Breast milk that was aseptically-collected from lactating mothers, who birthed at term, contained members of the *Lactobacillus*, *Streptococcus*, *Enterococcus*, *Peptostreptococcus*, *Staphylococcus*, *Corynebacterium*, and *Escherichia* species [78]. It can be argued that the *Escherichia* species, found specifically (IndVal = 0.95) in the six week old infant gut, may have originated from breast milk. This is consistent with the observed low abundance, and hence low transfer possibility, of this species from other maternal body sites (i.e., skin, nares, oral, vagina and stool) [27]. The microbial composition of breast milk varies among mothers in terms of beta diversity according to the time post-birth (e.g., ≤6 months after delivery or ≥6 months) [14]. By contrast, the alpha diversity varies with lifestyles (e.g., rural and urban) [85]. For example, the alpha diversity of the breast milk associated microbiota between rural and urban women showed significantly higher microbial diversity in the breast milk from rural women [85]. These differences in the alpha diversity of the breast milk associated microbiota from women with different lifestyles potentially provides for different seeding of the microbial population within the infants GI tract. A longitudinal study from Pannaraj et al. (2017) identified a stable alpha diversity within the breast milk microbiota (within sample diversity) in the first year of their babies’ lives. By contrast, the beta diversity (between samples diversity) of the breast milk microbiota increased during the first six months after birth, but slowly reduced when breast milk was no longer a primary source of nutrients [14]. 

Human milk oligosaccharides (HMOs) are the third largest component of breast milk. The types and amounts of HMOs produced from mothers who gave birth to preterm and term infants differ [90]. HMOs are a form of prebiotic and have been reported to be capable of promoting the growth of specific microorganism, including *Bifidobacteria* species [91,92] and *Bacteroidetes*, but not pathogenic bacteria, such as *Enterobacteriaceae* [92]. Although *Bifidobacteria* predominate in both formula- and breast-fed infants [37,79], the appearance in formula-fed infants is less frequent than that observed in breast-fed infants of the same age group [9,37,41,80,93,94]. Typically, infants will acquire a broad spectrum of *Bifidobacteria* species from the mother, but not all *Bifidobacteria* species are able to degrade HMOs [94]. Thus, strains of *Bifidobacteria* that are able to break down the individual specific HMOs that are present in the breast milk are most likely to predominate in the infant’s GI tract during early development [91,95]. A high lactose content and the presence of sialylated and fucosylated oligosaccharides in human breast milk, when compared to cow milk, can also promote the growth of *Bifidobacteria* over other bacteria [9]. 

### 5.3. The Effect of Antibiotic Usage on Gastrointestinal Microbial Development in Infants 

The use of antibiotics is more prevalent in infants born via C-section [39] and in those born preterm when compared to term infants born vaginally [96]. Maternal and infant exposure to antibiotics during the perinatal period has been linked to increased risks of later onset diseases, such as asthma [97], obesity [98], inflammatory bowel disease [99], and other allergic/inflammatory conditions [100] in children. Antibiotic exposure during the prenatal, perinatal, and postnatal periods has also been hypothesized to cause a delay in microbial maturation from 6 to 12 months after birth [101]. 

Intrapartum antimicrobial prophylaxis (IAP) is believed to be the most frequent source of antibiotic exposure in neonates [102]. IAP (penicillin, ampicillin, or ampicillin plus erythromycin) [103] is administered to mothers who are positive for group B *Streptococcus* during labour to reduce the risk of early-onset neonatal infections [62], such as pneumonia, septicaemia, and meningitis [104]. Two cohort studies of full term vaginally-born babies have reported reduced alpha diversity in faecal samples from infants that are exposed to maternal IAP when compared to non-IAP exposed infants [102,105]. Absolute levels of *Actinobacteria* and *Bacteroidetes* were lower in IAP-exposed infants in two different studies that performed 16s rRNA amplicon sequencing using the Illumina [102] and Ion Torrent [62] sequencing platforms. Significantly lower levels of *Bifidobacteriaceae* were also observed in IAP-exposed infants [62,102,105]. By contrast, the *Firmicutes* [102] and *Proteobacteria* [105] phyla increased numbers (*p* < 0.05 and *p* < 0.062, respectively) in IAP-exposed infants. 

Exposure of the maternal system to IAP impacts upon the early GI microbial composition in infants. However, the magnitude of the effect and its relationship to the duration (i.e., short- and long-term) and timing of the exposure is yet to be determined. Despite this, we know that the impact on certain bacterial counts within faecal samples from neonates in early life is affected by the combined effect of IAP exposure, postpartum feeding mode [105,106], and birth mode [107]. Studies conducted to date to understand the effects of these interactions on microbiota maturation have focused on term born babies in western countries [102,105,106,107]. Therefore, future studies should focus on the combined effects of these factors on GI microbial maturation processes, taking into account gestational age and the ethnicity of the infants.

### 5.4. Environmental Factors that Affect the Infants’ GI Microbiome Development 

Exposure to different extra uterine environments during early gut development contributes to the colonisation and evolution of the infant GI microbiota. Infants born by C-section are speculated to be more susceptible to environmental factors [20,91]. This is particularly true for preterm infants who have a higher chance of developing a flora that reflects the Neonatal Intensive Care Unit (NICU), due, in part, to the immaturity of their GIs and prolonged exposure to this environment [4]. 

The route of microbial transfer from the immediate environment to infants is hard to verify but studies have shown that microbes from the immediate environment can be isolated from infant faecal samples [17,108]. Cross-transmission between patients and dissemination of a multi-drug resistant (MDR) *Acinetobacter baumannii* strain also led to an outbreak in a NICU in Tunis. 31 neonates (gestational ages 26 to 41 weeks) developed MDR *A. baumannii* associated pneumonia and there were 10 deaths due to infection after the MDR *A. baumannii* was transferred from a neonate in the epidemic-associated surgical ward of another hospital [108]. These results are consistent with observations that infants from different geographical areas or different hospitals harbour different microbial populations [17,82]. However, further studies on larger groups of infants are required to verify the reliability and specificity of these results and to extend them to the population level. On the other hand, the PiPS trial, a double-blind randomised placebo-controlled trial of probiotic treatment with *Bifidobacterium breve* for the prevention of sepsis and necrotising enterocolitis in 1310 preterm babies born between 23 and 30 weeks’ gestation from 24 hospitals in the southeast England found that the probiotic strain of *Bifidobacterium breve* could be detected in the stool of 37% of babies in the placebo arm, as compared with 85% of the intervention arm, indicating that environmental-related factors resulted in significant cross-colonisation of *B. breve* in babies in this study [109]. Interestingly, this phase 3 PiPS trial also found no difference in the microbial diversity and richness of the microbiome of babies in the two arms of the study [110].

It is possible that the hospital environment, handling, feeding, and treatment regimes enhance microbial transmission to neonates [17]. However, the details of the mechanisms of transmission, dominant microbial populations within the hospital environments and the bacterial strains that have the highest chances to successfully colonise the infants’ GI remain elusive and are worth exploring in future studies.

#### Oxygen Levels in Term and Preterm Infants and Their Effects on GI Microbiota Development

The microbiota of children born preterm tend to more frequently contain detectable levels of pathogenic microorganisms (e.g., *K. pneumoniae* and *C. difficile*) [11,92,111], reduced microbial diversity [92,111], and contain low levels of short chain fatty acids (SCFA) [11]. Notably, when compared to term infants, preterm infants tend to be dominated by facultative anaerobes, including *Enterobacteriaceae* [112] and *Enterococcaceae* [5,41], and have low levels of anaerobes from the *Bifidobacterium*, *Bacteroides*, and *Atopobium* [5,41]. 

Newborn infants have an aerobic intestine at birth [113]. The high level of oxygen in the newborn GI tract favours the appearance of facultative anaerobes (e.g., *Enterobacteriaceae*, *Enterococcus*, and *Streptococcus*) [5,6,11,83,113,114]. Other facultative anaerobes are also present in the neonatal GI tract (e.g., *Staphylococci*, *Escherichia coli*, *Enterococcus fecalis*, and *faecium*, *Klebsiella*, *Enterobacter,* and, infrequently *Aeromonas*, *Pseudomonas*, and *Acinetobacter*) [3,6]. These early colonizers gradually create a reduced, anaerobic environment within the GI tract by consuming the available oxygen, consequently facilitating the establishment of obligate anaerobes (e.g., *Bifidobacterium*, *Clostridium*, *Bacteroides*, *Veillonella*, *Eubacterium*, and *Ruminococcus* species) [3,6,11,39,43,83,92,113]. In addition to reducing oxygen levels and facilitating the colonisation of the GI by strict anaerobes, many of the primary facultative anaerobic colonisers are potentially pathogenic [83,113].

Differences in oxygen levels exist between infants born term and preterm [115]. However, there is currently insufficient evidence to support oxygen levels as a factor contributing to the differences in the establishment of the gut microbiota in preterm infants. It remains likely that the observed differences in oxygen levels in the GI tract of preterm and term infants might be due to medical practices in the NICU. These differences could include the use of continuous positive airway pressure, which can lead to increased air in the preterm GI tract and can cause a delay in the establishment of the stage-specific commensal bacteria. Thus, it remains a possibility that GI oxygen levels contribute to health-related complications in preterm infants through a mechanism that enhances the levels of facultative anaerobes in the preterm gut. 

## 6. Conclusions

Gastrointestinal and gut microbiota maturation is an intricate, lengthy, and complicated process which starts in utero and continues after birth. Currently, there is no standardised definition of the composition of a “healthy” GI microbiota at different developmental stages and the main factors that contribute to the establishment of the GI microbiota in the early life remain elusive [63]. Seeding of the GI tract microbiome is suggested to begin in utero [23,68,116] and a representative of the intrauterine environment, meconium [13], was detected to harbour bacteria that have been detected in amniotic fluid [117]. GI microbial composition and species abundancy in infants are affected by variables that can directly or indirectly perturb the microbial community throughout the growth periods. Other than the apparent factors discussed in this review, other factors [10], such as geographical regions, host genetic factors, the effect of mix-feeding (breast milk and formula milk), hygiene level of healthcare providers, brands and content of formula milk consumed, and inter-individual variable are also likely to contribute to GI microbiota development. 

Albeit clear that environmental and extrinsic factors affect gut microbiota development, a lack of statistical power limits current studies with preterm infants. This is particularly relevant because nearly 15 million babies are born preterm each year worldwide and the rate of preterm birth is increasing [118]. Thus, there is a need for a powered longitudinal study that focuses on children born preterm and accounts for ethnicity and other potential confounders. 

Crosstalk between host cells (e.g., intestinal brush border cells or immune cells) and the colonising microbiota is likely to be critical for metabolic development and the programming of body immune system in infants [119,120]. Although the intrauterine environment has been proven to not be sterile, the influence of the maternal microbial community on the establishment of microbial population in utero is yet to be determined. As such, the studies will require experiments that highlight the pathways of bacteria translocation from the mother to the infants. Notably, the mechanisms of translocation during vaginal birth are still not fully understood, nor are the host characteristics that select the bacteria species that will be inherited from maternal gut. In order to fully understand how the GI microbiota, in its entirety, can be manipulated to enhance health, well-being and performance, it is essential to understand how each of these factors interact with one another to maintain intestinal homeostasis in developing infants, children, and adults. 

## Abbreviations

C-section	Caesarean section
DNA	Deoxyribonucleic acid
GI	Gastrointestinal
HMO	Human milk oligosaccharides
IndVal index	Indicator value index
IAP	Intrapartum antimicrobial prophylaxis
IBD	Inflammatory bowel disease
MDR	Multi-drug resistant
NICU	Neonatal Intensive Care Unit
RNA	Ribonucleic acid
SCFA	Short chain fatty acids
